# One-year mortality, quality of life and predicted life-time cost-utility in critically ill patients with acute respiratory failure

**DOI:** 10.1186/cc8957

**Published:** 2010-04-12

**Authors:** Rita Linko, Raili Suojaranta-Ylinen, Sari Karlsson, Esko Ruokonen, Tero Varpula, Ville Pettilä, the FINNALI study investigators

**Affiliations:** 1 Department of Anaesthesia and Intensive Care Medicine, Helsinki University Hospital, Sairaalakatu 1, PL 900, 00029 Helsinki, Finland; 2 Department of Intensive Care Medicine, Tampere University Hospital, Teiskontie 35, FI-33520 Tampere, Finland; 3 Division of Intensive Care, Kuopio University Hospital, Yliopistonranta 1E, FI-70211 Kuopio, Finland; 4 Australian and New Zealand Intensive Care Research Centre, School of Public Health, Monash University, The Alfred Hospital, Commercial Rd, Melbourne VIC 3004 Melbourne, Australia

## Abstract

**Introduction:**

High daily intensive care unit (ICU) costs are associated with the use of mechanical ventilation (MV) to treat acute respiratory failure (ARF), and assessment of quality of life (QOL) after critical illness and cost-effectiveness analyses are warranted.

**Methods:**

Nationwide, prospective multicentre observational study in 25 Finnish ICUs. During an eight-week study period 958 consecutive adult ICU patients were treated with ventilatory support over 6 hours. Of those 958, 619 (64.6%) survived one year, of whom 288 (46.5%) answered the quality of life questionnaire (EQ-5D). We calculated EQ-5D index and predicted lifetime quality-adjusted life years (QALYs) gained using the age- and sex-matched life expectancy for survivors after one year. For expired patients the exact lifetime was used. We divided all hospital costs for all ARF patients by the number of hospital survivors, and by all predicted lifetime QALYs. We also adjusted for those who died before one year and for those with missing QOL to be able to estimate the total QALYs.

**Results:**

One-year mortality was 35% (95% CI 32 to 38%). For the 288 respondents median [IQR] EQ-5D index after one year was lower than that of the age- and sex-matched general population 0.70 [0.45 to 0.89] vs. 0.84 [0.81 to 0.88]. For these 288, the mean (SD) predicted lifetime QALYs was 15.4 (13.3). After adjustment for missing QOL the mean predicted lifetime (SD) QALYs was 11.3 (13.0) for all the 958 ARF patients. The mean estimated costs were 20.739 € per hospital survivor, and mean predicted lifetime cost-utility for all ARF patients was 1391 € per QALY.

**Conclusions:**

Despite lower health-related QOL compared to reference values, our result suggests that cost per hospital survivor and lifetime cost-utility remain reasonable regardless of age, disease severity, and type or duration of ventilation support in patients with ARF.

## Introduction

Mechanical ventilation (MV) to treat acute respiratory failure (ARF) is common and it has been suggested that the use of these methods in ICUs will increase in the future [1]. Higher daily ICU costs are associated with the use of MV [2], so the amount of resources spent on this patient group is an important issue.

Along with a focus shift from short-term to long-term outcomes, the assessment of health-related quality of life (HRQOL) has gained more consideration in patients surviving after critical illness [3,4]. A combination of increased ICU costs and poorer long-term outcomes in patients with acute respiratory distress syndrome (ARDS) [5,6], the most severe form of ARF, and in patients needing prolonged MV [7-9], warrant calculations of cost-effectiveness and cost-utility analysis to be made based on HRQOL assessment.

Only two studies focusing on quality-adjusted life years (QALYs) after ARDS are available to date [5,10]. In addition, one cost-effectiveness study of MV [9] and few studies concerning subgroups of mechanically ventilated critical care patients have been published [11-14]. Thus, prospective large observational studies are needed to evaluate the outcomes and costs and predict the lifetime cost-utility of standard critical care in ARF.

Accordingly, we aimed to analyze one-year mortality and HRQOL of survivors and to predict lifetime QALYs gained and costs per QALY in a large prospective cohort, and relevant subgroups (such as non-invasive ventilation (NIV)), of consecutive ARF patients admitted to Finnish ICUs.

## Materials and methods

### ICUs and study population

We undertook a prospective multicenter cohort study (FINNALI) in 25 Finnish ICUs. These ICUs cover more than 97% of the adult population in Finland. During the eight-week period (between 16 April and 10 June, 2007), 2670 admissions in 25 Finnish ICUs were recorded. The cohort comprised all adult (≥ 16 years) patients (n = 958) who received invasive or non-invasive ventilatory support for more than six hours. NIV in this study comprised both continuous positive airway pressure (CPAP) and non-invasive positive pressure ventilation (NPPV). The epidemiology of ARF and the basic demographics of the FINNALI study have been reported previously [15]. This study is a prospective long-term follow up, QOL and cost-utility analysis of the FINNALI study.

### Data collection

Consent from the ethics committee was granted from each hospital. The ethics committees waived the need for informed consent for data registration. For one-year assessment of HRQOL we asked for a written consent. An EQ-5D questionnaire was mailed to patients who had consented. Permission to use the EQ-5D questionnaire was granted by the EuroQOL Group.

Patient characteristics including age, gender, prior functional status, admission reason, severity of acute illness (Simplified Acute Physiology Score (SAPS) II), organ dysfunction score (Sequential Organ Failure Assessment (SOFA)), resource use (Therapeutic Intervention Scoring 76 (TISS)) and length of ICU and hospital stay were obtained from the National ICU quality database (Intensium, Ltd, Kuopio, Finland). Activities of daily life were coded as: 1) able to work, 2) unable to work, but needs no help, 3) needs some help and 4) needs help with activities of daily life. Chronic health state and risk factors 48 hours before ARF were recorded in the clinical report form. The clinical report form data were combined with an internet-based interface to the quality consortium database.

### Outcomes

One-year mortality was obtained from Statistics Finland in 15 May, 2008 [16]. Follow-up time was calculated from the beginning of ARF.

HRQOL was assessed using the EQ-5D questionnaire, a standardized HRQOL instrument developed by the EuroQol Group, which has been found to be suitable and recommended for critically ill patients [3,10,17]. In the descriptive part of the EQ-5D, the respondents are asked to describe their health status for five dimensions: mobility, self-care, usual activities, pain/discomfort, and anxiety/depression. Each dimension is rated on a three-level scale: no, some, or severe problems. From the resulting five-digit EQ-5D health profile a weighted EQ index was calculated using the Finnish reference values [18]. Answers to all five domains were required for EQ-5D index calculation. For comparing values of respondents to Finnish population normals [18] the age of the respondent at admission to the ICU was used.

### Prediction of lifetime QALYs

We used the same method as the recently published study of conventional ventilation versus extracorporeal membrane oxygenation (CESAR) [10] for prediction of life time utility. For patients alive at the one-year follow up, the age- and gender-adjusted life expectancies from the year 2007 were obtained from Statistics Finland [16]. First, we predicted lifetime QALYs by multiplying predicted life expectancy after hospital survival by utility values (derived from the EQ-5D) for those survivors with utility values. Second, for patients who died during the one-year follow up, we used exact life-time after hospital discharge.

### Calculation of costs

Based on previous data [2] we reasoned that the mean ICU cost is inadequate for MV patients who consume more resources and individual TISS scores better reflect resource use. We, therefore, used number of individual TISS points for each patient during their ICU stay and the exact number of ward days for cost calculation. The average cost for one TISS-point (48€ per TISS point) was calculated by dividing the total annual cost of participating ICUs by the sum total of the annual TISS points. Mean ward day price was 416€ in Finnish hospitals [19]. Costs after hospital discharge were not available for evaluation. First, all costs (for non-survivors and survivors) were divided by the number of hospital survivors to calculate cost per hospital survivor. Second, the individual costs of all one-year survivors were divided by their predicted total lifetime QALYs to obtain a cost-utility value for only those with complete quality of life (QOL) data.

### Adjustment for missing data and sensitivity analysis

Finally, to confirm the robustness of our estimates we adjusted, first, for the patients who died before one year and, second, for those who did not respond to the QOL questionnaire using the mean QOL of age- and sex-matched respondents for QALY estimates, as previously published [19]. We assumed that non-survivors had lower QOL than the whole treated population. Thus, for those who died we used an estimate of 75% of QOL of the age- and sex-matched QOL of respondents and used the exact lifetime during the first year. For the one-year survivors with missing QOL we used the age- and sex-matched QOL of respondents after comparison to the QOL respondents. Thus, we calculated an estimate of the total predicted lifetime QALYs and cost per QALY for the entire population of 958 ARF patients of the FINNALI study.

### Statistical methods

Data are presented as medians and interquartile ranges (IQR), absolute values and percentages with 95% confidence intervals (CI) where appropriate, or means (SD). The two-tailed Mann-Whitney U test was used for comparison of continuous variables and the chi-squared test for categorical variables. Multiple groups were compared with Kruskal-Wallis test. EQ-5D index and reference values were analyzed with Wilcoxon's signed matched pair test. *P *value less than 0.01 was considered significant in all tests. SPSS 15.0 (SPSS inc., Chicago, IL, USA) was used for statistical analyses.

## Results

### All 958 ARF patients

**Figure 1:**
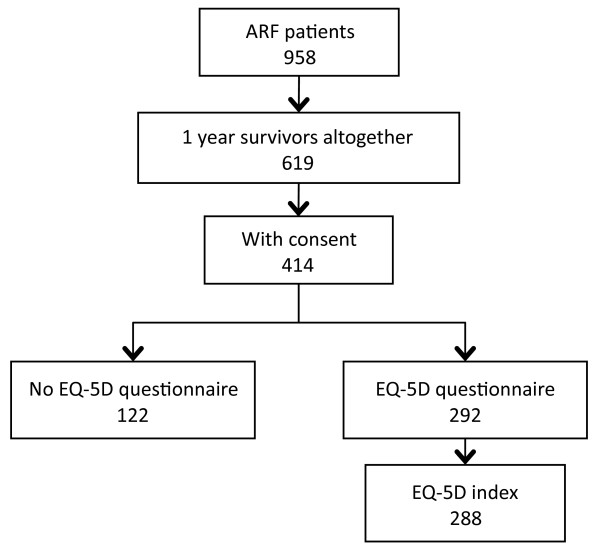
**Flow-chart of study population for quality of life and cost-utility evaluation**. ARF, acute respiratory failure; QALY, quality-adjusted life years.

A total of 958 patients were treated for ARF in ICUs during the study period. The flow chart of the study population is shown in Figure 1. Comparison of the characteristics of QOL respondents at one year (n = 288) and surviving non-respondents are compared in Table 1. Characteristics of all 958 ARF patients are also included in Table 1 for comparison. Half of the patients (482 of 949; 50.8%) were able to work before ARF. Only 1.8% (17 of 949) needed help in their daily activities. Daily activity data were missing for 9 patients.

**Table 1:** Demographic data of study patients. Data are presented as numbers (percentages) or median (interquartile range). Statistical significance was tested between respondents (n = 288) and non-respondents of quality of life questionnaire (EQ-5D) at one year

	ARF patients (n = 958)	One-year survivors (n = 619)		
			
		Respondents (n = 288)	Non-respondents (n = 331)	*P *value
Age, years	63 (51-74)	64 (53-74)	55 (41-66)	< 0.001
Gender, male n (%)	637 (66.5%)	195 (67.7%)	220 (66.5%)	0.74
SAPS II, points	43 (31-55)	37 (27-49)	37 (27-48)	0.99
SOFA, points	8 (6-10)	7 (5-9)	7 (5-9)	0.81
TISS, points, average	34 (29-41)	34 (29-43)	33 (28-39)	0.04
TISS, points, total	151 (90-280)	146 (95-271)	145 (78-264)	0.18
Operative n (%)	375 (39.1%)	143 (49.7%)	136 (41.1%)	0.03
Emergency n (%)	821 (85.7%)	217 (75.3%)	289 (87.3%)	< 0.001
Ventilatory support, days	2 (1-5)	2 (1-4)	2 (1-5)	0.81
ICU length of stay, days	3 (2-7)	3 (2-7)	3 (2-7)	0.29
Hospital length of stay, days	11 (6-21)	13 (8-23)	12 (7-23)	0.19
ALI/ARDS n (%)	68 (7.0%)	13 (4.5%)	20 (6.0%)	0.40

ALI, acute lung injury; ARDS, adult respiratory distress syndrome; ARF, acute respiratory failure; SAPS II, Simplified Acute Physiology Score; SOFA, Sequential Organ Failure Assessment; TISS, Therapeutic Intervention Scoring 76.

The one-year mortality rate was 35% (95% CI = 32 to 38%). Duration of MV was not associated with one-year mortality (*P *= 0.211). One-year mortality in the group with the shortest ventilatory support (less than 24 hours) was 32% (95% CI = 26 to 37%). The mortality (95% CI) in groups requiring ventilatory support 24 to 96 hours, 96 hours to 21 days, and over 21 days was 35% (30 to 40%), 40% (34 to 45%), and 31% (14 to 48%), respectively. However, a significant difference was found between the different ventilatory support groups. One-year mortality (95% CI) was lowest, 33% (95% CI = 30 to 36%), in patients treated with only invasive ventilation. In patients with NIV only, NIV failure during first six hours and NIV failure after six hours of treatment start were 37% (28 to 47%), 60% (45 to 76%) and 49% (31 to 66%), respectively (*P *= 0.001; 45% in all with NIV). One-year mortality in acute lung injury/ARDS patients was 51% (39 to 64%).

### The 288 one-year survivors with complete QOL data

Of the 292 returned EQ-5D questionnaires, 202 (69%) were given by patients, 42 (14%) by next of kin, and in 44 (15%) the respondent was unknown. The EQ-5D index could be calculated for 288 patients after one year (47% of 619 one-year survivors, and 67% of patients with consent). The only differences between the respondents and non-respondents were that the non-respondents were younger and more often patients with emergency admissions (Table 1).

**Figure 2:**
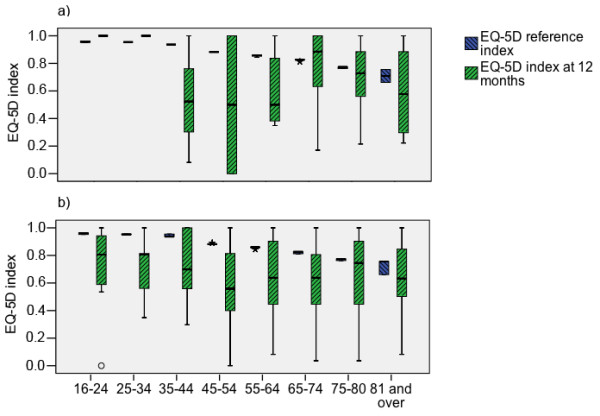
**EQ-5D index of respondents of acute respiratory failure at one year compared with reference values**. Patients are divided to (a) post-operative patients with short (< 1 day) ventilatory support and (b) other patients. *P *= 0.005 in age group 65 to 74 years.

The degree of impairment in each EQ-5D dimension is presented in Table 2. The EQ-5D index at one-year was lower than the age-matched and sex-matched reference value (0.70 (0.45 to 0.89) vs. 0.84 (0.81 to 0.88), *P *< 0.001). The EQ-5D index of the normal population declines with age, so one-year indexes are presented according to different age groups in Figure 2, separately for short postoperative patients (Figure 2a) and all other patients (Figure 2b). No significant difference in EQ-5D indices among one-year survivors was found between the age groups (*P *= 0.068 for men, *P *= 0.265 for women). However, significant differences were detected in all but the two oldest age groups when compared with the reference population. These 288 patients gained 6,583 life-years and 4,434 predicted lifetime QALYs (2,286 life-years and 1,540 QALYs per 100 one-year survivors). For these 288 one-year survivors the mean (SD) predicted life-years and lifetime QALYs were 22.9 (14.4) and 15.4 (13.3), respectively. The estimated total costs for these 288 were 4,830,402€ and 1,089€ per one predicted lifetime QALY.

**Table 2:** Distribution of responses to EQ-5D modalities at one year. Data are presented as number (percentage)

	Problems	n = 288
Mobility		
	No	120 (42%)
	Some	128 (44%)
	Severe	40 (14%)
Self-care		
	No	188 (65%)
	Some	75 (26%)
	Severe	25 (9%)
Usual activities		
	No	125 (45%)
	Some	113 (39%)
	Severe	50 (17%)
Pain/discomfort		
	No	86 (30%)
	Some	170 (59%)
	Severe	29 (11%)
Anxiety/depression		
	No	153 (53%)
	Some	121 (42%)
	Severe	14 (5%)

### Adjusted QOL and QALY for whole population

After adjustment for missing QOL assessments the median (IQR) sum index of EQ-5D for the total population was 0.60 (0.49 to 0.72). Accordingly, our 958 ARF patients were estimated to gain a total of 16,076 life years and 10,857 predicted lifetime QALYs (67 life-years and 44 QALYs in one year per 100 patients).

**Figure 3:**
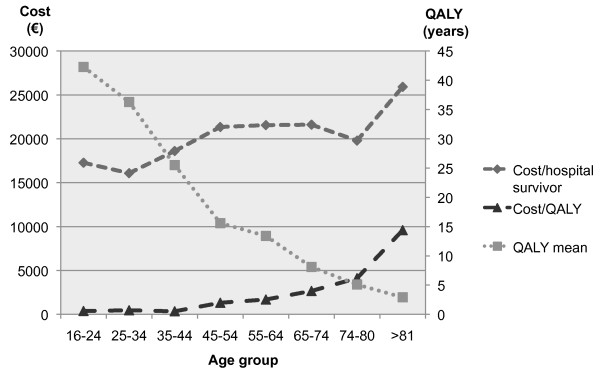
**Estimated mean costs and predicted lifetime quality-adjusted life years (QALY) and costs per QALY in different age groups**.

Overall the calculated costs (of survivors and non-survivors) for ICU and hospital stay were 20,739€ per hospital survivor. The proportion of ICU costs with regard to the total hospital cost (ICU and ward costs) were 73%, 69% and 87% for all, survivors and non-survivors, respectively (*P *< 0.001). For all 958 ARF patients (including non-survivors) the mean (SD) predicted life-years was 16.8 (17.2) and lifetime QALYs were 11.3 (13.0), respectively. Our calculation yielded a mean cost per lifetime QALY of 1,391€/QALY with a seven-fold range from 670€ to 5,263€ according to different age groups, preadmission status, admission type and disease severity (Table 3). When patients with short ventilation support (< 1day) after surgical procedure were excluded the mean cost per lifetime QALY for the rest of the patients was 1,483€/QALY. Estimated mean costs, predicted lifetime QALY and costs per QALY in different age groups of all ARF patients are presented in Figure 3. Both the cost per hospital survivor and the cost per lifetime QALY increased with the number of chronic illnesses and risk factors for ARF. Patients with unsuccessful NIV had the highest costs (42,625 to 44,971€ per hospital survivor). Individual costs and outcomes indicate that for 85% of our ARF hospital survivors the costs were lower than 20,000€ and for 88% they were lower than 50,000€ per predicted lifetime QALY. Among the patients with costs exceeding 20,000€, 27% (63 of 235) died before hospital discharge.

**Table 3:** Predicted cost-utilities in subgroups of patients with acute respiratory failure

	n	Gained survival (years)	QALYs (years)	Cost/hospital survivor	Cost/QALY
		mean (SD)	mean (SD)	€	€
**Age (years)**					
≤ 51	252	36 (19)	25 (16)	19,195	670
52-63	236	17 (11)	11 (8)	21,069	1,582
64-74	251	9 (8)	6 (6)	21,499	2,620
≥ 75	219	4 (5)	3 (3)	21,737	5,263
**SAPS II (points)**					
≤ 31	253	28 (18)	20 (15)	15,187	726
32-43	246	18 (16)	11 (12)	22,545	1,656
44-55	222	13 (15)	8 (11)	23,565	2,026
≥ 56	237	8 (13)	5 (9)	24,760	2,560
**Activities of daily life**					
Able to work	482	23 (19)	16 (14)	19,718	1,028
Unable to work but needs no help	337	12 (13)	8 (9)	21,665	2,075
Needs some help	113	8 (12)	5 (9)	21,512	2,722
Needs help to activities of daily life	17	8 (15)	5 (12)	30,653	3,450
**Admission type**					
Elective	133	16 (14)	12 (12)	17,273	1,283
Emergency	821	17 (18)	11 (13)	21,462	1,410
**Ventilatory support**					
NIV only	105	15 (17)	11 (14)	13,154	956
Invasive ventilation only	775	17 (17)	12 (13)	20,065	1,313
NIV and invasive ventilation before 6 hours	43	11 (16)	6 (9)	42,625	3,733
NIV and invasive ventilation after 6 hours	35	13 (17)	8 (11)	44,971	3,499
**Chronic diseases, count**					
0	425	23 (19)	16 (15)	19,065	966
1	320	13 (15)	8 (11)	22,583	1,936
2	158	10 (11)	7 (9)	21,699	2,337
3	47	9 (9)	6 (6)	24,400	3,102
4	8	8 (9)	4 (5)	34,108	3,910
**ARF risk factors 48 hours before**					
Sepsis	136	14 (15)	9 (12)	37,219	2,599
Cardiac insufficiency	192	9 (12)	6 (8)	27,322	2,779
Pneumonia	114	14 (16)	9 (12)	26,368	2,106
Post-operative with ventilatory support < 1 day	132	17 (15)	12 (12)	11,025	836

Chronic diseases include chronic obstructive pulmonary disease, chronic restrictive pulmonary disease, chronic heart disease, diabetes mellitus, immunodeficiency, neuromuscular disease.

ARF, acute respiratory failure; NIV, non-invasive ventilation; SAPS II, Simplified Acute Physiology Score; SD, standard deviation; QALY, quality-adjusted life year.

## Discussion

This prospective multicenter observational study of critically ill patients with ARF in 25 Finnish ICUs found that the 288 one-year survivors with complete QOL data were predicted to gain a mean of 22.9 life-years and 15.4 lifetime QALYs with a cost-utility of 1,089€ per lifetime QALY. After adjustment for missing values, the 958 ARF patients were estimated to gain a mean of 16.8 life-years and a mean of 11.3 predicted lifetime QALYs with a cost-utility of 1,391€ per one predicted lifetime QALY.

We used the EQ-5D as a measurement of HRQOL because it has been reported as suitable for critically ill patients [3], it may be answered reliably by the next of kin [20,21], and it has been previously used for cost-utility analysis in the critically ill [19,22,23]. More recently, a cost-utility evaluation alongside a large randomized trial comprising ARF and treatment with extracorporeal membrane oxygenation also used EQ-5D questionnaires (six months after critical illness) with UK tariff values for utility [10]. In concordance with our study, that study assumed a utility of 0 at the time of onset of treatment. This approach differs from cost-utility calculations for other medical treatment where a control group with no treatment is included. This kind of study design in ARF patients without MV would be unfeasible and unethical. Thus, an ordinary cost-utility analysis based on QOL comparison before and after treatment is not suitable in this specific setting of life-threatening illness where withdrawing MV means death. In the critical care setting, the ordinary concept of a control group seems to be only valid with regard to treatments, which are added on top of the vital life support such as MV, as in the CESAR study [10]. Accordingly, we did not include any comparisons to pre-ICU QOL but evaluated only QOL after critical illness for utility.

In agreement with previous studies [24-28] we detected lower QOL after ARF compared with the age-matched and sex-matched reference values. HRQOL decreases after ARF [29] and ARDS [5], and it may even be lower after ARDS compared with other critically ill patients [30]. Of note, the QOL after ARF may be largely influenced by severity of dysfunction and mortality rate.

The one-year mortality in this national cohort of ARF patients was 35%, which is below the previously reported range (range for one-year survival 44 to 65%) [5,7,29,31]. However, different definitions limit definite conclusions. The inclusion criteria for this study were a need for ventilatory support exceeding six hours using either invasive or non-invasive methods. Surprisingly, survival in the group that received the longest ventilatory support, over 21 days was as low as 31%, lower than the 51% and 58% in previous reports of prolonged MV [9,32]. Differences in decisions to withhold or withdraw treatments after a period of treatment effort may be one plausible explanation for this. In addition, the one-year mortality rate for those with NIV was 45% compared with 33% for those with invasive ventilation. Thus, studies in ARF patients including only patients with a special type or specified duration of MV may report varying survival rates. Thus, the varying inclusion criteria of different studies affect the predicted lifetime cost-utility.

Health care technologies that cost less than $20,000 per QALY are widely accepted, but even cut-off values as high as $100,000 per QALY have been considered [33]. The National Institute of Clinical Excellence (NICE) would be unlikely to reject any technology with a ratio in the range of £5,000 to £15,000 per QALY solely on the ground of cost-ineffectiveness [34]. Our sensitivity analysis (Table 3) showed that the cost remained below the lower cut-off value of NICE in all subsets. Thus, we consider our treatment cost-effective. Furthermore, compared with the Gross Domestic Product per capita (in Finland $37,200 2007 [35]) the cost-utility (as €/QALY) was lower in all subgroups of ARF patients. We detected remarkable differences in cost per QALY with increasing age and disease severity. Our finding is in parallel with the study by Hamel and colleagues in patients with pneumonia and ARDS [14]. However, in their study the cost range was considerably higher, from $19,000 to $200,000 per QALY according to different risk estimates and sensitivity analysis. Even without costs after discharge from hospital, hospital cost was higher than in our study. Even higher hospital costs ($165,075 to $423,596) have been reported for prolonged MV [9]. In addition, diagnostic category also influences cost among the critically ill [20,36,37]. Pneumonia and ARDS have been among the most expensive diagnosis groups in previous studies [14,35,38] and in a recent review [39]. In the absence of a gold standard for cost-effectiveness analysis, different ways to calculate costs and report the analysis make direct cost comparisons challenging [40].

To the best of our knowledge, this is the largest cost-utility analysis based on a nationwide prospective multicenter cohort of consecutive ARF patients. For QOL assessment we used EQ-5D, which is frequently used for QALY assessment in health care [41] and, despite its limitations, recommended for evaluations in the critical care setting [3], and reported recently in this setting with similar approach [10].

Our study has some limitations. First, we used an estimation of life expectancy for survivors to enable cost-utility over a lifetime horizon to be calculated, as previously published [10,19]. This approach includes an assumption that the life-time for survivors of ARF will be comparable with that of an age-matched and sex-matched population. We used one-year follow up instead of six months, as in the CESAR trial [10]. The survival after critical illness has been shown to be comparable with the Finnish population based reference values after two years [42]. However, we consider our follow up to be sufficiently long because mortality in our ARF patients was stable after six months, in keeping with previous reports [5,6,43]. Second, QOL was evaluated after one year and may not, even then, reliably reflect the QOL for the remainder of life. On the other hand, one-year follow up is generally accepted to be adequate [4,44]. Third, multiplying the life-time estimate by QOL at one-year follow up without adjusting for time differences does not take into account the gradual decline of QOL with age. However, we did not find any significant differences between the respondents of different age groups. In reference EQ-5D sum index values, the decline from the youngest to the oldest is 15 to 20% in the Finnish reference population. This decline would, therefore, correspond to a less than 10% increase in the estimated cost per QALY.

Fourth, all costs per QALY estimates are sensitive to differences in patient demographics and outcome. We, therefore, calculated costs separately for those with QOL assessment and also adjusted for patients with missing data. If we had only included one-year survivors, as some other studies with different critically ill patients [45], with QOL data available, that would have caused a significant bias because the surviving patients are not representative for the whole ICU population. We, therefore, also adjusted for missing patients with an approach similar to other studies [10,19,46] and performed a sensitivity analysis for different subgroups of patients ending up with acceptable estimates of up to 5,263€ per QALY for all these subgroups. Fifth, of note, these estimates do not include post-discharge costs or annual health service use as in some other studies [7,14]. Thus, our estimates only include hospital costs for the ARF treatment and underestimate the total costs of care during the first year.

Finally, precise costs of the care are difficult to gather and to compare with other studies because different calculations are used for ICU and hospital charges. We did not register the costs for each patient using a bottom-up method [40,47]. In order to be comparable with the latest Finnish study in severe sepsis [19] we used the same methodology for ICU and hospital costs adjusting for the use of ICU resources using individual TISS points for each patient in our calculations as previously published [48].

## Conclusions

We conclude that two-thirds of our ARF patients were alive at one-year follow up. Despite lower HRQOL compared with population reference value, our results suggest that the cost per one predicted lifetime QALY remains reasonable in these patients with ARF regardless of age, disease severity, or type and duration of MV.

## Key messages

• One-year mortality in ARF patients was 35% (95% CI = 32 to 38%).

• EQ-5D index after one year was lower than that of an age-matched and sex-matched general population.

• Cost per hospital survivor and life-time cost-utility remain reasonable regardless of age, disease severity, and type or duration of ventilatory support in patients with ARF.

## Abbreviations

ARDS: adult respiratory distress syndrome; ARF: acute respiratory failure; CI: confidence interval; CPAP: continuous positive airway pressure; HRQOL: health-related quality of life; IQR: interquartile range; MV: mechanical ventilation; NIV: non-invasive ventilation; NPPV: noninvasive positive-pressure ventilation; QALY: quality-adjusted life year; QOL: quality of life; SAPS: Simplified Acute Physiology Score; SD: standard deviation; SOFA: Sequential Organ Failure Assessment; TISS: Therapeutic Intervention Scoring 76.

## Competing interests

The authors declare that they have no competing interests.

## Authors' contributions

RL, TV, ER and VP were involved in the study design. RL and VP analyzed the data, made the statistical analysis and drafted the manuscript. SK, RSY, TV and ER participated in drafting and revision of the manuscript. All authors were involved in data acquisition and read and approved the final manuscript.
